# MR Angiography of Collateral Arteries in a Hind Limb Ischemia Model: Comparison between Blood Pool Agent Gadomer and Small Contrast Agent Gd-DTPA

**DOI:** 10.1371/journal.pone.0016159

**Published:** 2011-01-26

**Authors:** Karolien Jaspers, Bas Versluis, Tim Leiner, Petra Dijkstra, Marlies Oostendorp, Jolanda M. van Golde, Mark J. Post, Walter H. Backes

**Affiliations:** 1 Cardiovascular Research Institute Maastricht, Maastricht, The Netherlands; 2 Radiology, Maastricht University Medical Centre, Maastricht, The Netherlands; 3 Central Animal Facilities, Maastricht University, Maastricht, The Netherlands; 4 Internal Medicine, Maastricht University Medical Centre, Maastricht, The Netherlands; 5 Physiology, Maastricht University, Maastricht, The Netherlands; 6 GROW School for Oncology and Developmental Biology, Maastricht, The Netherlands; National Institutes of Health, United States of America

## Abstract

The objective of this study was to compare the blood pool agent Gadomer with a small contrast agent for the visualization of ultra-small, collateral arteries (diameter<1 mm) with high resolution steady-state MR angiography (SS-MRA) in a rabbit hind limb ischemia model. Ten rabbits underwent unilateral femoral artery ligation. On days 14 and 21, high resolution SS-MRA (voxel size 0.49×0.49×0.50 mm^3^) was performed on a 3 Tesla clinical system after administration of either Gadomer (dose: 0.10 mmol/kg) or a small contrast agent (gadopentetate dimeglumine (Gd-DTPA), dose: 0.20 mmol/kg). All animals received both contrast agents on separate days. Selective intra-arterial x-ray angiograms (XRAs) were obtained in the ligated limb as a reference. The number of collaterals was counted by two independent observers. Image quality was evaluated with the contrast-to-noise ratio (CNR) in the femoral artery and collateral arteries. CNR for Gadomer was higher in both the femoral artery (Gadomer: 73±5 (mean ± SE); Gd-DTPA: 40±3; *p*<0.01) and collateral arteries (Gadomer: 18±4; Gd-DTPA: 9±1; *p* = 0.04). Neither day of acquisition nor contrast agent used influenced the number of identified collateral arteries (*p* = 0.30 and *p* = 0.14, respectively). An average of 4.5±1.0 (day 14, mean ± SD) and 5.3±1.2 (day 21) collaterals was found, which was comparable to XRA (5.6±1.7, averaged over days 14 and 21; *p*>0.10). Inter-observer variation was 24% and 18% for Gadomer and Gd-DTPA, respectively. In conclusion, blood pool agent Gadomer improved vessel conspicuity compared to Gd-DTPA. Steady-state MRA can be considered as an excellent non-invasive alternative to intra-arterial XRA for the visualization of ultra-small collateral arteries.

## Introduction

Therapeutic stimulation of the development of collateral arteries from pre-existent arterioles (arteriogenesis) seems attractive as an alternative or adjuvant treatment for patients with peripheral arterial occlusive disease [1], [2], [3]. Further progress in the development of such treatment strategies relies strongly on the availability of non-invasive imaging methods that are able to evaluate the efficacy of treatment in terms of vascular changes at an early stage, even before clinical benefit can be noticed.

Therapeutic efficacy can be evaluated functionally, i.e. by measuring perfusion recovery-related parameters in tissue distal to the vascular lesion, or morphologically, i.e. by quantifying the number and size of the collaterals formed. Contrast-enhanced MR angiography (CE-MRA) has already proven to be a promising non-invasive tool to visualize collateral arteries in various parts of the body [4], [5], [6], [7]. Preferably, CE-MRA images are acquired during the first pass of the bolus, when the arterial concentration is highest and veins are not yet enhanced. However, visualization of small peripheral collateral arteries (diameter<1 mm) requires both a very high spatial resolution and a large spatial coverage. Meeting these requirements within the duration of the first pass period is a challenge. Moreover, correct timing of the first-pass acquisition is problematic in patients with severe stenoses and an extensive network of collateral arteries, whose filling occurs very slowly and varies considerably among and within patients [8], [9].

An alternative is to acquire data during the post-bolus equilibrium phase or steady state, which permits longer acquisition times and therefore a higher spatial resolution. Due to its longer acquisition time, steady-state MRA (SS-MRA) is less sensitive for timing uncertainties than first-pass MRA (FP-MRA), as demonstrated for the visualization of arterial stenoses in patients with peripheral arterial occlusive disease [10]. To obtain sufficient signal from the arteries, a high arterial contrast agent concentration over the entire duration of the acquisition is desired. However, most currently available contrast agents are small (e.g. gadopentetate dimeglumine (Gd-DTPA), molecular weight: 0.5 kDa) and extravasate rapidly after injection, resulting in both a decrease in arterial signal intensity and an increase in background enhancement. These problems may be solved by the use of larger contrast agents which remain mainly intravascular, thereby providing prolonged vascular enhancement [11]. Moreover, these so-called blood pool agents (BPAs) generally have a higher *T*_1_ relaxivity compared to small contrast agents because of their size [12], allowing reduction of contrast agent dose without compromising signal-to-noise characteristics [13], [14], [15], [16], [17].

Various types of types of gadolinium-based BPAs have been developed. One group consists of small BPAs that non-covalently bind to plasma proteins, with the albumin-binding gadofosveset trisodium as its principal, clinically approved example [18]. A second group consists of medium-sized blood pool agents such as P792 [19], [20] and Gadomer [21], which are currently under consideration for use in humans. Their value in diagnostics and pretreatment assessment of peripheral and coronary arterial disease has been demonstrated [10], [11], [22], [23], [24], yet their potential in the visualization of ultra-small arteries required for the evaluation of collateral artery formation remains to be explored.

In this study, we compared the blood pool agent Gadomer to the small contrast agent Gd-DTPA for the visualization of small collateral arteries with steady-state contrast-enhanced MRA in a standardized rabbit hind limb ischemia model exhibiting arteriogenesis. For both contrast agents, image quality characteristics and number of visible collaterals were determined and compared to intra-arterial XRA.

## Materials and Methods

### Animal model

This study was approved by the Maastricht University animal ethics committee (approval ID 06-022). Ten male New Zealand White rabbits (weight: approximately 3 kg) were included. Occlusion of the right femoral artery was realized by placing ligations 1 cm below the branch of the circumflex femoral artery (CFA) and 1 cm above the bifurcation of the popliteal and tibial arteries.

For all procedures, anesthesia was induced by intramuscular injection of a combination of ketamine hydrochloride (50 mg/kg body weight) and xylazin (8 mg/kg body weight). During the procedures, the rabbits were intubated and ventilated with isoflurane (2–3%) and oxygen. Post-operative analgesia was provided during three days by daily intramuscular injections of buprenorphine (0.09 mg/kg body weight). All invasive procedures were performed under sterile conditions. After the last MR measurements a lethal dose of pentobarbital (1 mL/kg body weight) was administered intravenously.

### X-ray angiography

On days 14 and 21 post ligation, XRA series of anterior-posterior projections were recorded using a portable x-ray system (BV Pulsera, Philips Medical Systems, Best, The Netherlands). In-plane resolution was 0.2×0.2 mm, tube voltage was 72 kV and frame rate was 12 s^−1^. An intra-arterial catheter was inserted into the left carotid artery and positioned in the iliac artery of the ligated limb, where a 5 mL bolus of non-ionic iodine contrast agent (Omnipaque, Amersham Health, Eindhoven, The Netherlands, 240 mg iodine/mL) was injected at 5 mL/s, followed by a saline flush.

### MR angiography

Within one hour after XRA, the animals were imaged in supine position on a clinical 3.0 Tesla MRI system equipped with a 5-element phased-array cardiac coil (Philips Medical Systems, Best, The Netherlands). The rabbits were placed in a custom-made fixating box to standardize animal positioning.

The steady-state MRA (SS-MRA) protocol consisted of a *T*_1_-weighted spoiled fast gradient echo sequence. Field-of-view (FOV) in the cranio-caudal read-out direction was 250 mm and rectangular FOV was 80%. Parallel imaging acceleration (SENSE) factor in the right-left direction was 2. Acquisition was started after a delay of two minutes. Matrix size was 512×410 with 282 overlapping coronal slices (slice thickness 0.5 mm). Measured voxel size was 0.49×0.49×0.50 mm^3^. TR/TE/FA were 10.5 ms/2.7 ms/25°. Acquisition time was 5 minutes.

### Contrast agents

To all animals both a small extravascular agent (Gd-DTPA) and a medium-sized blood pool agent (Gadomer) were administered. Gadomer (Schering Pharma AG, Berlin, Germany) is a dendritic gadolinium chelate, containing 24 Gd^3+^ ions. It has a molecular weight of 17 kDa. Gd-DTPA and Gadomer were administered in a randomized order on separate days. The contrast agent (vial concentration 0.5 M, injected volume approximately 0.3 mL or 0.6 mL, for Gadomer and Gd-DTPA, respectively) was injected at 0.05 mL/s into an ear vein, followed by 2 mL saline flush injected at the same rate.

The contrast agent dose was selected by estimating signal enhancement during the course of SS-MRA acquisition based on a biexponential approximation of contrast agent concentration determined in a previous study [25] for a range of doses. A full description of the procedure is found in Appendix S1; see also Figure S1. The optimal dose for Gadomer was 0.10 mmol Gd/kg. For Gd-DTPA, maximum signal enhancement was obtained with a dose of 0.50 mmol Gd/kg. However, this was considered too high, and the difference among doses in the range of 0.20–0.50 mmol Gd/kg was small. We therefore selected the lowest dose in this range (0.20 mmol/kg). For these doses, the average concentration during image acquisition is 0.8 mM for Gadomer and 1.3 mM for Gd-DTPA.

### Image analysis

#### Image processing

From the dynamic XRA image series a maximum intensity projection (MIP) was calculated in MATLAB (The MathWorks, Natick, MA, version R2007b) over the time frames that exhibited arterial enhancement. The resulting images were used for collateral identification. The MR angiograms were viewed in the image processing application OsiriX (version 3.7), using the source images for signal intensity measurements. For collateral identification, coronal maximum intensity projections over a limited range of slices (partial MIPs) were used. The thickness and location of the slab, as well as the display contrast and brightness levels could be adjusted in real time to obtain optimal depiction of the collateral trajectories and varied depending on image quality and vascular anatomy.

#### Collateral quantification

The number of collaterals was counted on the partial MIP by two independent observers using the Longland definition [26], which requires identification of the stem, mid- and re-entry zone. A distinction was made between collaterals stemming from the deep femoral artery (DFA) and CFA [27]. To evaluate whether the same collateral arteries were identified between measurements, the observers provided a schematic representation of the course of the identified collaterals, including the approximate position of the stem and re-entry point.

Arteries were separated from veins based on their size, location, and branching point. Due to evident differences in image quality, the observers were not blinded for contrast agent.

#### Image quality

Signal intensities (SI) were measured in the femoral artery (proximal of the occlusion), a collateral artery stemming from the DFA, and in muscle tissue surrounding the collateral. Noise was estimated by calculating the standard deviation of a relatively homogeneous part of adjacent muscle tissue (SD_muscle_). From these data signal-to-noise (SNR) and contrast-to-noise (CNR) ratios were calculated according to:
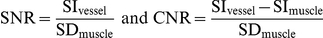



### Statistics

Interobserver agreement of the collateral artery counting was assessed with a between-observer coefficient of variation. The difference in number of collaterals between XRA and MRA was tested using a paired Wilcoxon signed rank test. A repeated analysis of variance (ANOVA) was used to test whether day of acquisition or contrast agent used (for MRA) had an influence on the number of identified collateral arteries. Independent sample two-sided Student's *t*-tests were performed to test the difference in SNR and CNR between Gadomer and Gd-DTPA for the femoral artery and a collateral stemming from the DFA in ligated limb. Effects were considered significant for *p*<0.05.

## Results

### Image quality

**Figure 1:**
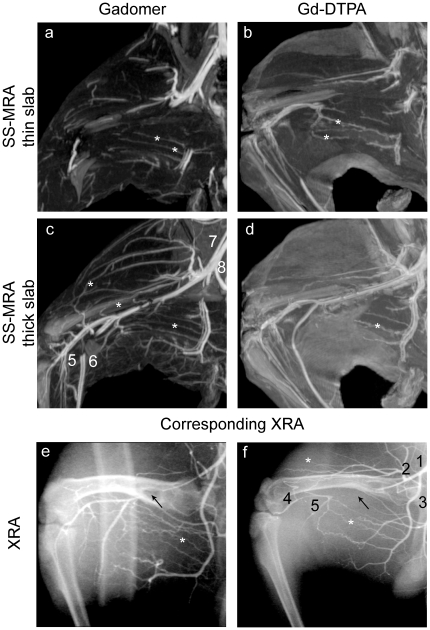
Contrast-enhanced MR angiograms with Gadomer and Gd-DTPA. Partial maximum intensity projections (MIPs) in the anterior-posterior direction of steady-state MR angiograms (SS-MRA) for Gadomer (left column) and Gd-DTPA (right column). On the thin-slab MIPs (thickness 3–4 slices, panels a and b), collaterals could be discerned for both contrast agents. Thick slab MIPs (>20 slices; panels c and d) show superior depiction of vessels with Gadomer. For Gd-DTPA, the images were blurred. Panels e and f show the corresponding x-ray angiograms (XRA). 1: femoral artery; 2: circumflex femoral artery; 3: deep femoral artery; 4: tibial artery; 5: popliteal artery; 6: popliteal vein; 7: iliac artery; 8: iliac vein. * indicate collaterals. The ligation is indicated with the arrow in the XRA images. All images were acquired on day 21.

On the steady-state MR angiograms, both arteries and veins were enhanced, but the spatial configuration of the vessels and the high spatial resolution of the angiograms allowed distinction of arteries from veins (see Figure 1 and Videos S1 and S2). For Gadomer, thick-slab MIPs (thickness>10 mm) provided the best overview of the vascular system, but for Gd-DTPA these images appeared too blurred to be of use. On thin-slab MIPs (thickness<5 mm), the advantage of Gadomer was less pronounced. Although the femoral and collateral arteries appeared more conspicuous with Gadomer, contrast between artery and background tissue was sufficiently high to identify the complete trajectories of the collaterals for both contrast agents.

**Figure 2:**
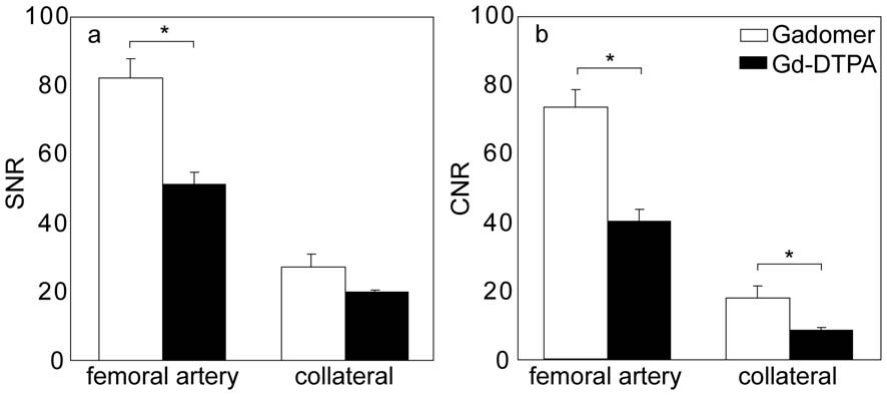
SNR and CNR in femoral and collateral artery. Mean signal-to-noise (SNR, panel a) and contrast-to-noise ratio (CNR, panel b) in the femoral artery and a collateral artery originating from the deep femoral artery in the steady-state MR angiograms for Gadomer (white) and Gd-DTPA (black). Error bars indicate standard error of the mean. Significant differences between contrast agents (*p*<0.05) are indicated with *. Data were averaged over days 14 and 21 post ligation.

SNR was higher in both the femoral and collateral arteries for Gadomer compared to Gd-DTPA (Figure 2). This improvement was significant in the femoral artery (*p*<0.01), and had the same, yet statistically non-significant trend in the collateral arteries (*p* = 0.10). CNR was significantly higher in both femoral and collateral arteries when Gadomer was used (femoral artery: *p*<0.01; collateral artery: *p* = 0.04). ROI size was typically 10 voxels in the femoral artery and 3 voxels in the collateral artery. Noise and background signal was measured over an area of approximately 3 cm^2^.

### Collateral quantification

Table 1 lists the results of the collateral quantification. On day 14, an average of 4.9 and 4.3 collaterals was found for Gadomer and Gd-DTPA, respectively, and on day 21 an average of 5.8 (Gadomer) and 4.5 (Gd-DTPA) arteries were identified. Analysis of variance revealed that neither day of acquisition nor contrast agent used influenced the number of identified collateral arteries (*p* = 0.30 and *p* = 0.14, respectively). Although the animals were positioned in a custom-made fixating box, it was not feasible to perform 1-to-1 comparison of the collaterals found with either contrast agent due to difficulties in determining the exact course of the collateral arteries. Inter-observer variation in the number of identified collaterals on SS-MRA was 24% and 18% for Gadomer and Gd-DTPA, respectively, meaning that the difference between observers is approximately one collateral artery. Identification of the exact points of branching and re-entering appeared to be highly subjective, and no conclusions could be drawn from the schematic vessels courses.

**Table 1:** Number of identified collateral arteries.

	Day 14	Day 21
Gadomer	4.9±1.1	5.8±1.3
Gd-DTPA	4.3±0.9	4.5±1.0
XRA	5.1±1.7	6.3±1.8*

Number of identified collaterals (mean ± SD) on steady-state MR angiograms (MRA), averaged over two observers, with Gadomer and gadopentetate dimeglumine (Gd-DTPA) and intra-arterial x-ray angiograms (XRA) on days 14 and 21.

*indicates an significant increase in number of collaterals (p<0.05).

The number of collateral arteries found with XRA increased between days 14 and 21 (day 14: 5.1±1.7 (mean ± SD); day 21: 6.3±1.8; *p* = 0.04). The number of collateral arteries found with XRA was equal to the number found with MRA (*p* = 0.08). For XRA, most collaterals originated from the deep femoral arteries (120 out of 172 or 70%), whilst for MRA only 50% (77 out of 153) stemmed from the DFA.

## Discussion

### Current findings

To our knowledge, this is the first study that directly compares the efficacy of the blood pool agent Gadomer with Gd-DTPA for the visualization of ultra-small collateral arteries. The number of collaterals identified on the steady-state images was equal for the two contrast agents, and closely mirrored the number of collaterals identified on high resolution invasive X-ray angiograms. Contrast enhancement in collateral arteries in terms of CNR and therefore vessel conspicuity, was superior for Gadomer compared to Gd-DTPA.

### Blood pool agents versus small contrast agents

The use of Gadomer resulted in a considerably higher contrast between collateral arteries and background compared to Gd-DTPA in the SS-MRA images. Because of this improved image quality, thick-slab MIPs could be used to get a better overview of the vessel trajectories, which notably facilitated the localization of collateral arteries, thus making it less time-consuming. For Gd-DTPA, the value of these thick-slab MIPs was limited, because background enhancement became problematic for slabs thicker than approximately 8 slices (5 mm). Moreover, thick-slab MIPs may suggest connections between vessels which are in fact crossings of different vessels. Therefore, to follow the trajectory of the collateral artery from stem to re-entry zone, thin-slab MIPs were used, which combined the favorable contrast-to-noise characteristics of source images with the improved overview related to MIPs. Using thin-slab MIPs for the actual identification diminished the disadvantage for Gd-DTPA, as the contrast between collaterals and background was still high enough to identify an equal number of collaterals compared to XRA, leaving little room for improvement with Gadomer.

One of the main advantages of blood pool agents is their decreased extravasation. For Gadomer, the extravasation rate is approximately 10 times lower compared to Gd-DTPA [28], which has two positive effects for angiography. First, a slower decrease in blood concentration during the distribution phase could be observed, as demonstrated in a previous study [25]. Second, the lower extravasation rate resulted in decreased contrast agent concentrations in the background tissue [28]. To obtain the same vascular contrast enhancement, the dose of Gadomer could be reduced by a factor 2 compared to Gd-DTPA due to a higher *r*_1_ value for Gadomer.

Although Gadomer has already been safely used for angiography in humans [22], it is not yet approved. The potential advantage of Gadomer over the clinically approved BPA gadofosveset trisodium is that it is remains intravascular due to its intrinsic structure rather than to its binding affinity with albumin. During first-pass, a large (74% [17]) fraction of gadofosveset trisodium is not yet bound and extravasates more rapidly, as gadofosveset trisodium has a size comparable to Gd-DTPA. Moreover, at 3 Tesla, compared to gadofosveset, Gadomer has a higher *r*_1_ (Gadomer: 13 L mmol^−1^ s^−1^; gadofosveset: 9.9 L mmol^−1^ s^−1^) and a lower *r*_2_ (Gadomer: 25 L mmol^−1^ s^−1^; gadofosveset: 60 L mmol^−1^ s−^1^), which may favor Gadomer for imaging at 3 Tesla.

### MRA versus XRA

In addition to the advantages regarding invasiveness and ionizing radiation, MRA has the advantage of the availability of 3D data compared to 2D XRA, which is a one-directional projection. On these projections no distinction can be made between arteries that branch off or cross other arteries, which hinders the identification of stem and re-entry zones. A second disadvantage of XRA is the high signal from bone structures. In clinical practice, this problem is usually solved by using digital subtraction of pre- and post-contrast images. In our study, vasospasms and animal movement due to contrast injection regularly resulted in blurred images which were less suited for collateral identification. Consequently, the proximity of the femur to the mid and re-entry zone of collaterals stemming from the circumflex femoral artery resulted in the identification of fewer collaterals stemming from the CFA on XRA images compared to MRA. Although the spatial resolution achieved with MRA is generally lower than with XRA, MRA compared well to XRA for both contrast agents and the total number of collaterals visualized on MRA was comparable to that of XRA.

### Conclusion

The blood pool agent Gadomer has great potential in the visualization of small collateral arteries with steady-state contrast-enhanced MRA. The total number of identified collaterals was equal to XRA. Although no difference between Gadomer and Gd-DTPA in number of identified collaterals or interobserver variability was found in this study, the enhanced collateral artery conspicuity appreciably facilitated the identification of collateral arteries with Gadomer compared to Gd-DTPA, while the dose was reduced.

## Supporting Information

Figure S1 **Signal enhancement time course.** Signal enhancement time course for a range of contrast agent doses for Gadomer (panel a; range: 0.05–0.20 mmol/kg) and Gd-DTPA (panel b; range 0.10–0.50 mmol/kg). Grey shaded regions indicate the acquisition window for steady-state MRA in this study.
(TIF)

Appendix S1 **Dose optimization.** Procedure for the selection of the optimal dose for Gadomer and Gd-DTPA.
(DOC)

Video S1 **MRA with Gadomer.** Dynamic overview of a typical MRA data set with Gadomer. Partial maximum intensity projections in the anterior-posterior direction of steady-state MR angiograms (SS-MRA) were obtained at varying positions. Slab thickness was 2 cm.
(AVI)

Video S2 **MRA with Gd-DTPA.** Dynamic overview of a typical MRA data set with Gd-DTPA. Partial maximum intensity projections in the anterior-posterior direction of steady-state MR angiograms (SS-MRA) were obtained at varying positions. Slab thickness was 2 cm.
(AVI)
