# Supplementary figures and images for: MR Angiography of Collateral Arteries in a Hind Limb Ischemia Model: Comparison between Blood Pool Agent Gadomer and Small Contrast Agent Gd-DTPA
Source: PLoS One. 2011 Jan 26;6(1):e16159. doi: 10.1371/journal.pone.0016159 (PMC3027628; doi:10.1371/journal.pone.0016159)

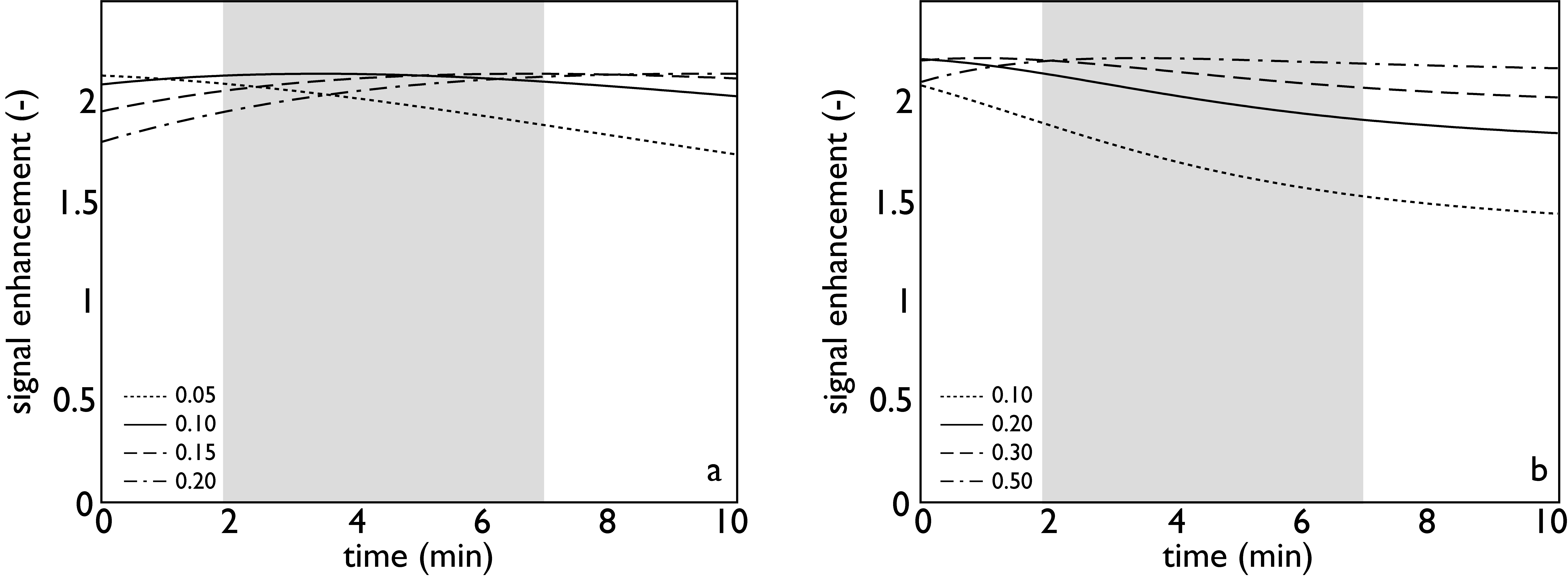

Supplement: Figure S1 — Signal enhancement time course. Signal enhancement time course for a range of contrast agent doses for Gadomer (panel a; range: 0.05–0.20 mmol/kg) and Gd-DTPA (panel b; range 0.10–0.50 mmol/kg). Grey shaded regions indicate the acquisition window for steady-state MRA in this study. (TIF) [file pone.0016159.s001.tif]
