# Supplementary material for: MR Angiography of Collateral Arteries in a Hind Limb Ischemia Model: Comparison between Blood Pool Agent Gadomer and Small Contrast Agent Gd-DTPA
Source: PLoS One. 2011 Jan 26;6(1):e16159. doi: 10.1371/journal.pone.0016159 (PMC3027628; doi:10.1371/journal.pone.0016159)
Supplement: Appendix S1 — Dose optimization. Procedure for the selection of the optimal dose for Gadomer and Gd-DTPA. (DOC) [file pone.0016159.s002.doc]

Appendix S1: Dose optimization

### **Methods**

The optimal dose for steady-state MRA was determined for each contrast agent based on their pharmacokinetic properties in plasma for rabbits, and the computed relation between contrast agent concentration and signal enhancement. The plasma concentration time curve C_p_ in rabbits can be described by a biexponential decay function:

(1)

where *D* is the administered dose (unit: mmol/kg). Amplitudes *A_1_* and *A_2_* and time constants τ_1_ and τ_2_ were determined by blood sampling in the femoral artery, as described in a previous experiment [1]. *A*_1_ and *A*_2_ were found to be 22 and 9.7 kg/L for the SCA and 24 and 5.5 kg/L for the MCA. τ_1_ and τ_2_ were 2.5 and 75 min for the SCA and 5.5 and 70 min for the MCA. C_p_ was converted to the whole blood contrast agent concentration C_b_(t) by correcting with the hematocrit value (Hct = 0.40 [1]).

Concentrations were converted into *T*_1_ and *T*_2_^*^ using values from literature for pre-contrast *T*_1_ and *T*_2_^*^ of blood and muscle tissue [2], and *r*_1_ and *r*_2_^*^. *T*_2_ and *r*_2_ were used as approximations for *T*_2_^*^ and *r*_2_^*^. For Gadomer, plasma *r*_1_ and *r*_2_ were 13 and 25 L mmol^-1^ s^-1^ [3] at 3 Tesla, respectively. For Gd-DTPA, *r*_1_ is 3.7 and *r*_2_ is 5.3 L mmol^-1^ s^-1^. Based on these *T*_1_ and *T*_2_ values, signal amplifications were calculated using a formula for signal intensity in a spoiled fast gradient echo sequence [4]. Signal enhancement was defined as the ratio between post- and pre-contrast signal intensity.

### Results

Figure S1 shows the signal enhancement time course for a range of contrast agent doses for Gadomer (panel a; range: 0.05 – 0.20 mmol/kg) and Gd-DTPA (panel b; range 0.10 – 0.50 mmol/kg). The optimal dose for Gadomer was 0.10 mmol Gd/kg. For Gd-DTPA, maximum signal enhancement was obtained with a dose of 0.50 mmol Gd/kg, although the difference with doses in the range of 0.2-0.5 mmol Gd/kg was small. We therefore selected the lowest dose in this range (0.20 mmol/kg).

1. Jaspers K, Aerts HJ, Leiner T, Oostendorp M, van Riel NA, et al. (2009) Reliability of pharmacokinetic parameters: small vs. medium-sized contrast agents. Magn Reson Med 62: 779-787.

2. Stanisz GJ, Odrobina EE, Pun J, Escaravage M, Graham SJ, et al. (2005) T1, T2 relaxation and magnetization transfer in tissue at 3T. Magn Reson Med 54: 507-512.

3. Rohrer M, Bauer H, Mintorovitch J, Requardt M, Weinmann HJ (2005) Comparison of magnetic properties of MRI contrast media solutions at different magnetic field strengths. Invest Radiol 40: 715-724.

4. Hendrick R, Roff U (1991) Image contrast and noise. In: Stark D, Bradley W, editors. Magnetic Resonance Imaging. Chicago: Moshby Yearbook. pp. 135.
